# Outcomes and Healthcare Resource Utilisation in Adults With von Willebrand Disease Treated With Recombinant von Willebrand Factor in Surgical Settings in the United Kingdom

**DOI:** 10.1111/ejh.70033

**Published:** 2025-10-11

**Authors:** Mike Laffan, Heena Howitt, Cheryl Jones, Sarah Brighton, Rosa Willock, Anna Sanigorska, Oliver Heard

**Affiliations:** ^1^ Centre for Haematology, Imperial College London Hammersmith Hospital London UK; ^2^ Takeda UK Ltd. London UK; ^3^ HCD Economics Knutsford UK

**Keywords:** adult, delivery of health care, health resources, operative, retrospective studies, surgical procedures, treatment outcome, United Kingdom, von Willebrand diseases, von Willebrand factor

## Abstract

**Objectives:**

We describe treatment outcomes and healthcare resource utilisation (HCRU) in adults with von Willebrand disease (VWD) receiving recombinant von Willebrand factor (rVWF) in surgical settings in the United Kingdom.

**Methods:**

Retrospective chart review of adults (≥ 18 years) with congenital VWD receiving first‐time rVWF for the prevention/treatment of surgery‐related bleeds, or the on‐demand treatment of spontaneous/traumatic bleeds, between 1 October 2020 and 30 June 2022 at seven hospitals. Treatment, outcome and VWD‐related HCRU data associated with the prevention/treatment of surgery‐related bleeds were collected at first (index) event, for 12 months pre‐index and 3–12 months post‐index.

**Results:**

Twenty patients (55.0% female, 90.0% White/Caucasian, mean age 56.7 years) received rVWF to prevent/treat surgery‐related bleeds at index: normal haemostasis following abnormal bleeding was achieved for all applicable bleeds with limited requirement for additional treatments. Only four patients (all VWD type 2) received exogenous factor VIII in addition to rVWF at index. There were no treatment switches and no likely treatment‐related complications. Physician‐rated treatment satisfaction was ‘excellent’ (36.7%) or ‘good’ (63.3%) for all rVWF‐treated surgery‐related bleeds (*n* = 30).

**Conclusions:**

Results support the effectiveness and safety profile of rVWF in a surgical setting in adults with VWD, supplementing the growing body of evidence for rVWF.

## 1 Introduction

Von Willebrand disease (VWD) is considered the most common inherited bleeding disorder, occurring among men and women equally, although women are more likely to notice the symptoms that affect them, such as heavy or abnormal bleeding during menstrual periods and after childbirth [1]. According to the United Kingdom (UK) National Haemophilia Database, it was estimated that 16.5 per 100 000 people in the UK had VWD (all types) in 2020 (type 1: 7.2 per 100 000 people; type 2: 2.5 per 100 000; type 3: 0.3 per 100 000; remaining patients ‘unclassified’ or ‘low von Willebrand factor’ [VWF]) [2].

Bleeding complications in patients with VWD vary depending on age, sex, disease type and level of VWF activity [3]. Patients with VWD most often experience bleeding after surgery or trauma and mucosa‐associated bleeding (e.g., epistaxis, menorrhagia or oral/gum bleeding) [3, 4, 5]. Poorer health‐related quality of life and greater healthcare resource utilisation (HCRU) have been reported for patients with VWD than for general populations [6, 7, 8, 9, 10, 11].

In the UK, recombinant von Willebrand factor (rVWF; vonicog alfa) is currently indicated for surgical bleeding or the prevention and treatment of haemorrhage in adults with VWD, when desmopressin (DDAVP) alone is ineffective or contraindicated [12]. rVWF has been shown to be efficacious for the peri‐operative and on‐demand management of bleeding in patients with VWD in Phase 3 clinical trials [13, 14]. It was authorised in August 2018, but real‐world data describing its use and treatment outcomes in patients with VWD in clinical settings in the UK are limited. Furthermore, there is a paucity of real‐world evidence (RWE) describing the HCRU of patients with VWD who are treated with rVWF in the UK.

This UK retrospective chart review evaluated real‐world experience with rVWF used for the prevention and resolution of bleeds during surgery, or on demand to treat spontaneous or traumatic bleeds. The objective of this analysis is to describe treatment outcomes and HCRU in adults with VWD treated with rVWF for the prevention and/or treatment of bleeds in surgical settings. Outcomes and HCRU associated with the on‐demand use of rVWF to treat spontaneous or traumatic bleeds in adults with VWD have been reported in a separate publication [15].

## 2 Methods and Materials

### 2.1 Study Design

A retrospective chart review study was conducted on adults with congenital VWD at seven UK Haemophilia Comprehensive Care Centres. Patients were enrolled at the time of their first rVWF administration (index event) between 1 October 2020 and 30 June 2022 (index period) (Figure S1A). Patients who had surgery may have received rVWF as prophylaxis in preparation for surgery and/or as treatment on the day of surgery (pre‐, intra‐ and/or post‐operatively) and/or as prophylaxis to prevent post‐operative bleeding in the days following surgery, as deemed necessary by the treating physician (Figure S1B).

Patient data were collected from medical records by physicians using an electronic case report form at first rVWF administration (index event), for 12 months before the index event (pre‐index) and until death, loss of follow‐up, or end of study 3–12 months after the index event (post‐index). This study was conducted in accordance with Good Clinical Practice. Full ethical approval was obtained from the Yorkshire & Humber—Leeds West Research Ethics Committee and patient informed consent was collected prior to enrolment.

### 2.2 Patient Population

Eligible patients with a diagnosis of congenital VWD must have received their first ever administration of rVWF within its licensed indication (as at study commencement) [16] during the index period; been at least 18 years of age at their index event; and had at least 3 months of follow‐up after their index event.

Patients were excluded if they had other bleeding disorders or factor deficiency (including acquired von Willebrand syndrome), VWF neutralising antibodies/inhibitors, or had participated in clinical trials during the study period. Two adults, one who underwent minor surgery and one with a muscle haematoma at index, were eligible for inclusion in the study but have been excluded from this analysis because they received long‐term VWF prophylaxis during the study period.

### 2.3 Objectives

The primary objective for this analysis was to describe the real‐world first‐time use of rVWF in the prevention and/or treatment of surgery‐related bleeds at index, including subsequent bleed‐related outcomes, in adults with congenital VWD. The secondary objectives were to describe VWD‐related HCRU associated with all surgery‐related bleeds recorded during the study period, and the surgery type, severity and category, treatment and duration, and related outcome(s) of all surgery‐related bleeds during the 12 months prior to and the 3–12 months following the first rVWF administration.

Exploratory objectives included describing differences in bleed‐related outcomes and HCRU for index, pre‐ and post‐index surgery‐related bleeds treated with rVWF only, rVWF plus another agent, other factor treatments, non‐haemostatic treatments and treatment involving a switch. HCRU by inpatient or outpatient visit was also described by pooling surgery‐related bleeds across index, pre‐ and post‐index periods in patients treated with or without rVWF. Finally, treatment combinations stratified by prophylaxis in preparation for surgery, on the day of surgery and prophylaxis following surgery were described for surgeries at index, pre‐ and post‐index.

### 2.4 Assessments

The number, type (major, minor, dental or trauma, as categorised by the physician), severity and category (elective vs. emergency) of surgeries for which rVWF was administered as prophylaxis or on the day of surgery were assessed. The number of patients treated with rVWF prophylactically in preparation for surgery or to prevent post‐operative bleeding in the days following surgery, and on the day of surgery, was also described. Further data on the prevention or treatment of surgery‐related bleeds, including the number of infusions received by the patient, average dose/kg, total dose received and treatment duration for rVWF plus any other drugs used (e.g., plasma‐derived [pd] VWF concentrates, factor VIII [FVIII] products, DDAVP and tranexamic acid [TXA]) were collected.

Achievement of normal haemostasis after abnormal bleeding related to surgery, that is, bleed control and resolution, was described (where applicable, according to the opinion of the treating physician), along with information on treatment switches, including the reason for switching. Post‐operative bleeds (within 14 days of operation) or rebleeds (worsening of condition either on treatment or within 72 h of stopping treatment for a post‐operative bleed) were described by the treating physician as mild, moderate or severe.

Physician‐assessed satisfaction with rVWF treatment was collected using a 4‐point rating scale (‘excellent’, ‘good’, ‘moderate’ or ‘poor’). Adverse events and treatment‐emergent adverse events (TEAEs) were collected using Common Terminology Criteria for Adverse Events (CTCAE) grading version 5.0 (mild, moderate, severe, life‐threatening and death) [17].

The VWD‐related HCRU data collected comprised the number of patients requiring inpatient and intensive care unit [ICU] admissions, length of stay in hospital (including in ICU) and number of outpatient visits (day cases).

### 2.5 Statistical Analyses

All data were summarised descriptively. Categorical variables were presented as frequencies and percentages; continuous variables were summarised as means with standard deviation (SD). Endpoints were reported separately for the index event and any pre‐index or post‐index events during the follow‐up period. Descriptive statistics for treatment, outcomes and HCRU associated with rVWF‐treated surgeries were reported for stratifications by VWD type and surgery type (minor, major or dental‐related). Analyses were performed using STATA 17 (StataCorp. 2021. Stata Statistical Software: Release 17. College Station, TX: StataCorp LLC).

## 3 Results

**FIGURE 1:**
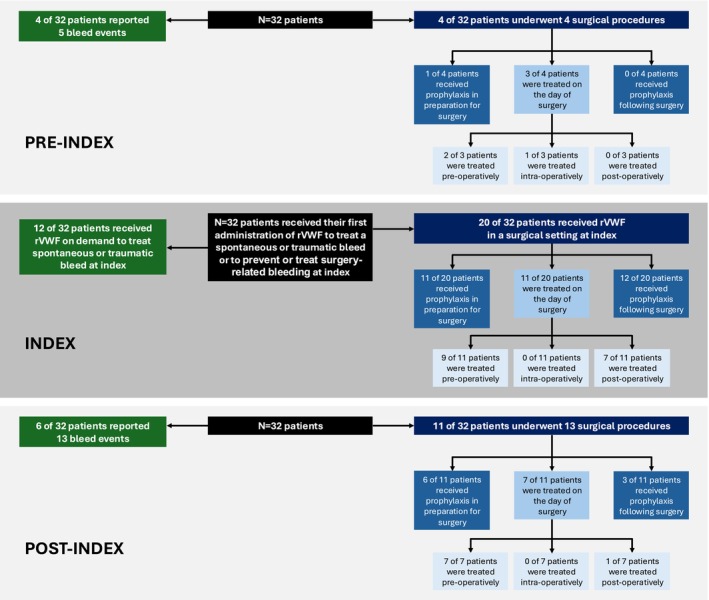
Patient flow diagram: Index and pre‐/post‐index (*N* = 32). rVWF, recombinant von Willebrand factor.

In total, 32 patients received rVWF to prevent or treat a surgery‐related bleed or treat a spontaneous or traumatic index bleed at index (Figure 1 and Table S1).

### 3.1 Prevention and/or Treatment of Surgery‐Related Index Bleeds With rVWF

#### 3.1.1 Demographics and Baseline Characteristics of Patients

Overall, rVWF was used to prevent or treat surgery‐related index bleeds in 20 patients with a mean (SD) age at index of 56.7 (18.8) years and a mean (SD) time since diagnosis of 25.2 (17.1) years. Approximately half (55.0%) of these patients were female and 90.0% were White/Caucasian (Table 1). The mean (SD) weight and body mass index (BMI) of these patients were 83.3 (20.3) kg and 28.7 (5.3) kg/m^2^, respectively. The majority of patients (90.0%) had type 1 (*n* = 7) or type 2 (*n* = 11) VWD, with two patients unclassified. Twelve patients (60.0%) had a familial history of VWD and 12 had ≥ 2 comorbidities at index. Laboratory test values at presentation by VWD type and by surgery type are reported in Table S2.

**TABLE 1:** Demographics and baseline characteristics of adults with VWD treated with rVWF at index in a surgical setting.

Variable	*N* = 20
Age at index (years), mean (SD)	56.7 (18.8)
Female, *n* (%)	11 (55.0)
White/Caucasian, *n* (%)	18 (90.0)
Weight (kg), mean (SD)	83.3 (20.3)
BMI (kg/m^2^), mean (SD)	28.7 (5.3)
VWD type, *n* (%)	
Type 1	7 (35.0)
Type 2A	3 (15.0)
Type 2B	3 (15.0)
Type 2M	4 (20.0)
Type 2N	1 (5.0)
Type 3	0
Unclassified	2 (10.0)
Time since diagnosis (years), mean (SD)	25.2 (17.1)
Familial history of VWD, *n* (%)	12 (60.0)
Missing data, *n* (%)	7 (35.0)
Family member with VWD, *n* (%)^a^	
Parent	9 (75.0)
Sibling	7 (58.3)
Grandparent	1 (8.3)
Other	4 (33.3)
Laboratory test values at presentation (IU/mL),^b^ mean (SD)	
VWF:RCo (*n* = 15)	0.6 (0.6)
FVIII:C (*n* = 17)	1.0 (0.7)
VWF:Ag (*n* = 17)	0.9 (0.8)
Comorbidities at index,^c^ *n* (%)	
0	5 (25.0)
1	3 (15.0)
2	3 (15.0)
> 2	9 (45.0)

*Note: N* = number of patients.

Abbreviations: Ag, antigen; BMI, body mass index; FVIII, factor VIII; RCo, ristocetin cofactor; rVWF, recombinant von Willebrand factor; SD, standard deviation; VWD, von Willebrand disease; VWF, von Willebrand factor.

^a^ Percentage calculated with number of patients with non‐missing data in denominator.

^b^ Not all lab tests were performed at presentation on all patients. Mean (SD) is reported only for patients on whom each test was performed (*n* = x).

^c^ Within 2 years of data abstraction.

#### 3.1.2 Bleed Characteristics and Treatment

**FIGURE 2:**
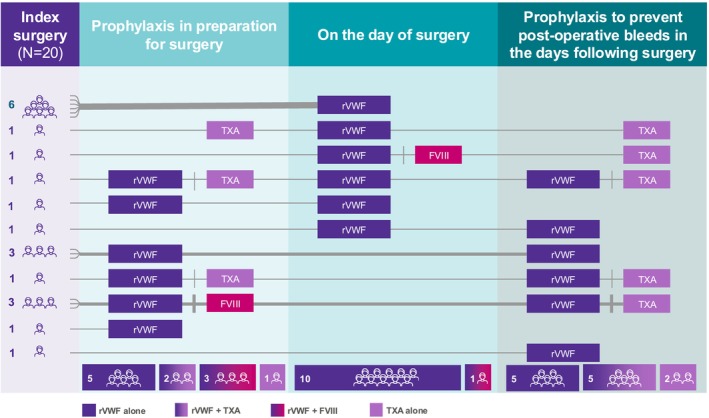
Treatment and prevention of surgery‐related index bleeds in adults with VWD (*N* = 20). FVIII, factor VIII; rVWF, recombinant von Willebrand factor; TXA, tranexamic acid.

In total, 14 patients (70.0%) received surgery‐related prophylaxis with any treatment or combination of treatments: 11 (55.0%) received prophylaxis in preparation for surgery and 12 (60.0%) received prophylaxis following surgery. Eleven patients were treated with rVWF on the day of surgery; one of these patients (VWD type 2A) also received FVIII for a minor surgery (Figures 1 and 2).

Twelve patients (60.0%) underwent major surgery (one related to trauma) and eight (40.0%) underwent minor surgery (none were trauma‐related). Most (95%) surgeries were elective, and all bleeds were classified by the treating physician as non‐life threatening. Three patients had a post‐operative bleed, all related to major surgery: two were moderate and one was mild.

Ten patients (50.0%) received rVWF as prophylaxis in preparation for surgery with a mean (SD) of 1.1 (0.3) infusions, a mean (SD) dose per infusion of 44.4 (17.8) IU/kg, a mean (SD) total consumption of rVWF of 3510.0 (2171.0) IU and a mean (SD) treatment duration of 1.1 (0.3) days (Table 2). Ten patients received rVWF as prophylaxis to prevent post‐operative bleeding in the days following surgery, with a mean (SD) of 6.3 (5.6) infusions. The mean (SD) dose per infusion was 23.0 (10.2) IU/kg, the mean (SD) total consumption was 8130.0 (3770.8) IU and the mean (SD) treatment duration was 5.8 (4.4) days.

**TABLE 2:** Treatment and prevention of surgery‐related bleeds at index in adults with VWD treated in a surgical setting.

Variable	Prophylaxis in preparation for surgery (*n* = 11)	As needed on the day of surgery (*n* = 11)	Prophylaxis to prevent further bleeds in the days following surgery (*n* = 12)
Treatment, *n* (%)			
rVWF only	5 (45.5)	10 (90.9)	5 (41.7)
FVIII concomitantly with index rVWF dose	3 (27.3)	1 (9.1)	—
TXA concomitantly with index rVWF dose	2 (18.2)	—	5 (41.7)
TXA only	1 (9.1)	—	2 (16.7)
rVWF dosing (IU), mean (SD)	3055.0 (1063.7)	2835.6 (945.6)	1613.0 (528.6)
Number of infusions	1.1 (0.3)	2.1 (1.4)	6.3 (5.6)
Dose per infusion (IU/kg)	44.4 (17.8)	31.6 (9.5)	23.0 (10.2)
Total consumption (IU)	3510.0 (2171.0)	5627.3 (3160.9)	8130.0 (3770.8)
Duration of treatment (days)	1.1 (0.3)	1.9 (0.9)	5.8 (4.4)
FVIII dosing (IU), mean (SD)	1833.3 (577.4)	3000.0 (0.0)	—
Number of infusions	1.0 (0.0)	1.0 (0.0)	—
Dose per infusion (IU/kg)	29.4 (8.3)	27.8 (0.0)	—
Total consumption (IU)	1833.3 (577.4)	3000.0 (0.0)	—
Duration of treatment (days)	1.0 (0.0)	1.0 (0.0)	—
TXA dosing (mg), mean (SD)	1000.0 (0.0)	—	1000.0 (0.0)
Number of infusions	1.7 (1.2)	—	30.7 (17.9)
Dose per infusion (mg/kg)	14.0 (2.1)	—	14.3 (3.0)
Total consumption (mg)	1666.7 (1154.7)	—	30714.3 (17904.5)
Duration of treatment (days)	1.0 (0.0)	—	8.9 (4.3)

Abbreviations: FVIII, factor VIII; rVWF, recombinant von Willebrand factor; SD, standard deviation; TXA, tranexamic acid; VWD, von Willebrand disease.

Of the 11 patients treated with rVWF on the day of surgery, nine were treated pre‐operatively and seven were treated post‐operatively, with an overall mean (SD) of 2.1 (1.4) infusions, a mean (SD) dose per infusion of 31.6 (9.5) IU/kg, a mean (SD) total consumption of rVWF of 5627.3 (3160.9) IU and a mean (SD) treatment duration of 1.9 (0.9) days.

#### 3.1.3 Bleed‐Related Outcomes

Of the 11 patients described above who received rVWF on the day of surgery (either post‐operatively or pre‐ and post‐operatively), eight (72.7%) achieved normal haemostasis following abnormal bleeding during surgery. The other three patients were only treated pre‐operatively and experienced no abnormal bleeding. There were no reported treatment switches among these 11 patients. For all 20 surgical cases using rVWF at index, the treating physician rated treatment satisfaction as ‘excellent’ (50.0%) or ‘good’ (50.0%) irrespective of the time of administration.

#### 3.1.4 Safety

Three adverse events (AEs) related to index surgery‐related bleeds were reported during the study period: one thrombotic event following knee surgery; one urinary retention secondary to a clot; and one cardiovascular event following elective eye surgery. All were considered mild. The urinary retention secondary to a clot was in a patient aged over 65 years who had been discharged while taking TXA. The patient who experienced a cardiovascular event (hypertension) was over 80 years of age and had a history of hypertension, atherosclerotic heart disease and hypercholesterolemia. Neither event was considered related to rVWF by the treating physician. A distal deep vein thrombosis (DVT) identified 7 days after total knee‐replacement surgery in a patient aged over 85 years was interpreted as unlikely to be related to rVWF by the treating physician. The patient presented with VWF ristocetin cofactor and FVIII concentration levels of 0.26 and 0.91, respectively, and received a combination of treatments pre‐surgery, on the day of surgery and up to 14 days post‐surgery (including rVWF, rFVIII and TXA). The patient did not receive post‐operative thromboprophylaxis or anticoagulant treatment for the DVT, but wore intermittent compression devices on legs after surgery and continued rVWF treatment. There was no extension of DVT and no clot on repeat ultrasound. No allergies or anaphylaxis were reported in the study.

### 3.2 Prevention and/or Treatment of Pre‐ and Post‐Index Surgery‐Related Bleeds

#### 3.2.1 Bleed Characteristics and Treatment

Pre‐index, that is, during the 12 months prior to first rVWF administration for index event, four of the 32 patients in the study underwent surgery (Figure 1): all were elective, one was major surgery, one was minor and two were dental‐related. All related bleeds were classified as non‐life‐threatening. One patient who had dental‐related surgery had a mild post‐operative bleed. In this period, one patient received prophylaxis with DDAVP in preparation for surgery, and three patients were treated on the day of surgery (two pre‐operatively, one with human VWF and one with pdVWF/FVIII complex and one intra‐operatively with TXA) (Table S3).

Post‐index, that is, during the 3–12 months after first rVWF administration for index event, 11 of the 32 patients in the study had 13 surgeries (Figure 1), seven of which (53.9%) were minor, three were major and three were dental‐related surgeries. Twelve of the 13 surgeries were elective; only one emergency surgery took place post‐index. All surgery‐related bleeds (where information was available) were classified as non‐life‐threatening, with information missing for one surgery. There were no post‐operative bleeds or rebleeds related to surgeries recorded post‐index. Six patients received prophylaxis in preparation for surgery post‐index; two received rVWF only, one received rVWF and TXA, two received pdVWF/FVIII complex and TXA and one received DDAVP and TXA. Seven patients were treated with rVWF on the day of surgery, six pre‐operatively only and one pre‐ and post‐operatively. Three patients received prophylaxis to prevent further bleeds in the days following surgery, two each with rVWF and TXA and one with pdVWF/VIII complex.

#### 3.2.2 Bleed Outcomes

Pre‐index, normal haemostasis was achieved after abnormal bleeding with all products used on the day of surgery, with no reported treatment switches. Post‐index, all seven patients who received rVWF on the day of surgery achieved normal haemostasis after abnormal bleeding, with no reported treatment switches.

The treating physicians rated treatment satisfaction with rVWF for all post‐index surgery‐related bleeds (*n* = 10) as ‘excellent’ (10.0%) or ‘good’ (90.0%).

### 3.3 VWD‐Related HCRU of Adults Treated for Surgery‐Related Bleeds

An overview of VWD‐related HCRU for surgery‐related bleeds during all periods is presented in Table 3. For surgery‐related treatment at index, five patients (25.0%) had an outpatient visit only. Fourteen patients (70.0%) had an inpatient admission, only two of which included admissions to an ICU. One patient (5.0%) had both an inpatient admission and an outpatient visit. The mean (SD) length of inpatient stay was 8.2 (10.3) days, with a mean (SD) of 4.5 (2.1) additional days for those admitted to ICU during their stay.

**TABLE 3:** VWD‐related HCRU of adults treated for surgery‐related bleed events.

Variable	Pre‐index *N* = 4 surgeries	At index *N* = 20 surgeries	Post‐index *N* = 13 surgeries
Number of adults with an inpatient admission, *n* (%)	0 (0.0)	15 (75.0)^a^	4 (30.8)^a^
Length of inpatient stay (days), mean (SD)	—	8.2 (10.3)	3.3 (2.9)
Number of adults with an ICU admission,^b^ *n* (%)	0 (0.0)	2 (10.0)	0 (0.0)
Length of ICU stay (days), mean (SD)	—	4.5 (2.1)	—
Number of adults with an outpatient visit, *n* (%)	4 (100.0)	6 (30.0)^a^	10 (76.9)
Number of outpatient visits per patient, mean (SD)	1.0 (0.0)	1.2 (0.4)	1.1 (0.3)

Abbreviations: HCRU, healthcare resource utilisation; ICU, intensive care unit; rVWF, recombinant von Willebrand factor; SD, standard deviation; VWD, von Willebrand disease.

^a^ One patient with an inpatient admission also had an outpatient visit.

^b^ Adults with an ICU admission are a subset of adults with an inpatient admission.

### 3.4 Exploratory Outcomes

#### 3.4.1 VWD‐Related HCRU for Pooled Surgery‐Related Bleeds Treated With and Without rVWF by Type of Hospitalisation

**FIGURE 3:**
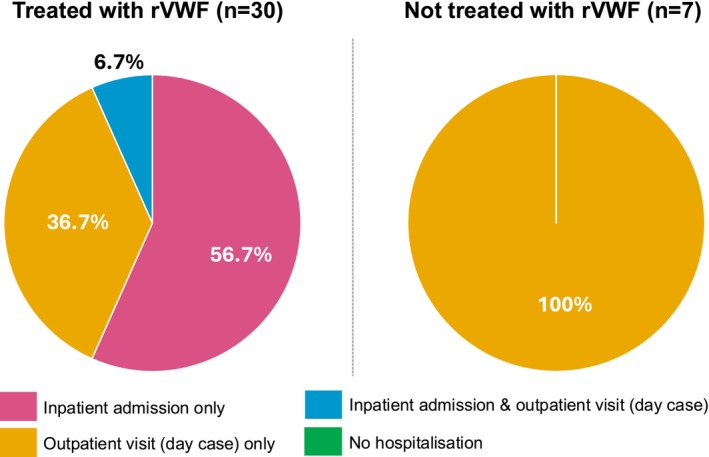
HCRU associated with treatment for surgery with and without rVWF* in adults with VWD (pooled across index, pre‐ and post‐index). *Bleeds treated without rVWF may have been treated with pdVWF/FVIII complex, pdVWF/FVIII complex + TXA, human VWF, DDAVP, DDAVP + TXA, or TXA. DDAVP, desmopressin; HCRU, healthcare resource utilisation; pd, plasma‐derived; rVWF, recombinant von Willebrand factor; TXA, tranexamic acid; VWD, von Willebrand disease; VWF, von Willebrand factor.

When pooling surgery‐related bleeds across index, pre‐index (when rVWF treatment was not possible by definition) and post‐index, 30 of a total of 37 surgeries (81.1%) were treated with rVWF (of which 50.0% were major surgeries) and seven (18.9%) were treated with products not including rVWF (of which one was associated with major surgery) (Figure 3).

The mean (SD) total consumption of rVWF on the day of surgery for patients admitted as inpatients (5345.0 [3284.0] IU) was higher than that for those with outpatient visits (3818.8 [2209.4] IU). However, the mean (SD) dose per kg per patient was lower for inpatients (27.6 [6.0] IU/kg) compared with outpatients (34.5 [13.5] IU/kg).

#### 3.4.2 Characteristics, Treatment, Outcomes and HCRU of rVWF‐Treated Surgery‐Related Bleeds Stratified by VWD Type and by Surgery Type

The surgery type most frequently associated with VWD type 2 patients was major surgery (53.3%), followed by minor surgery (33.3%). In type 1 patients, minor surgery was most frequent (54.6%) and in those with unclassified VWD type, major and minor surgeries were reported equally (each 50.0%).

rVWF was used as prophylaxis in preparation for surgery in the type 1 (*n* = 3), type 2 (*n* = 9) and unclassified groups (*n* = 1) with mean (SD) doses per infusion of 39.9 (13.3), 45.7 (16.4) and 17.8 IU/kg, respectively. rVWF was used as prophylaxis to prevent post‐operative bleeding in the days following surgery in one patient each with type 1 and unclassified VWD (doses of 29.7 and 17.8 IU/kg, respectively) and in 10 type 2 patients (mean [SD] dose: 22.6 [11.1] IU/kg). The mean (SD) rVWF dose per infusion used pre‐, intra‐ or post‐operatively on the day of surgery was highest in patients with type 1 VWD (34.8 [8.2] IU/kg; *n* = 9), followed by patients with type 2 disease (28.8 [10.3] IU/kg; *n* = 6) and patients in the unclassified group (22.0 [12.8] IU/kg; *n* = 3).

Major surgeries (including one trauma treated with major surgery) were most commonly treated with rVWF (50.0% of all procedures), followed by minor surgeries (43.3%) and dental surgeries (6.7%). rVWF was used as prophylaxis in preparation for major surgery (*n* = 1), minor surgery (*n* = 5) and dental surgery (*n* = 1); mean (SD) doses per infusion were 48.5 (18.8), 36.1 (10.8) and 29.2 IU/kg, respectively. rVWF was used as prophylaxis following major surgery (*n* = 8) and minor surgery (*n* = 4), with mean (SD) doses per infusion of 25.1 (11.0) and 18.1 (8.2) IU/kg, respectively. The mean (SD) duration of rVWF prophylaxis following surgery varied between major and minor surgeries, with 7.0 (6.0) and 5.8 (3.8) infusions over 6.5 (4.5) and 3.8 (2.1) days, respectively.

The mean (SD) dose of rVWF per infusion used pre‐, intra‐ or post‐operatively on the day of surgery was highest for dental (*n* = 1) and minor (*n* = 8) surgeries at 34.2 and 33.5 (13.8) IU/kg, respectively, compared to major surgery (27.8 [6.3] IU/kg; *n* = 9).

Inpatient admissions were required for all major surgeries and for 30.8% of minor surgeries. All dental surgeries were performed in the outpatient setting.

## 4 Discussion

This chart review provides real‐world evidence of the efficacy of rVWF for the treatment and prevention of surgical bleeding in line with data from a clinical trial [14], which demonstrates the replicability of these outcomes. To our knowledge, this is the largest UK‐based study to report treatment outcomes and HCRU in adults with VWD treated with rVWF for the prevention and/or treatment of bleeds in surgical settings. Overall, the results of the study showed that rVWF is effective for the prevention and treatment of surgery‐related bleeds in adults with type 1 or type 2 VWD. The limited use of additional agents alongside rVWF to prevent or resolve bleeding further highlighted its effectiveness. Two‐thirds of patients with surgery‐related bleeds at index were treated with rVWF alone and the remainder were treated with rVWF in combination with another agent, of which only one (5.0%) patient (VWD type 2A) was treated with FVIII and rVWF, both given on the day of minor surgery. Similarly, a minority of patients were treated with FVIII and rVWF as prophylaxis in preparation for surgery (two with VWD type 2A and one with type 2N, all for major surgeries). Notably, all patients treated to prevent or resolve surgery‐related bleeds during the post‐index period in this study were treated with rVWF.

The real‐world evidence reported here also supports the safety profile of rVWF, with only three complications reported (one thrombotic event, one urinary retention secondary to a clot and one cardiovascular event), all of which were mild and unlikely to be related to, or not related to, rVWF treatment. Furthermore, physician‐rated treatment satisfaction was rated as ‘excellent’ or ‘good’ for all surgery‐related bleeds at index and post‐index for which rVWF was used.

These findings are aligned with those of a similar study conducted using the same methodology across Europe (Austria, Denmark, France, Germany, the Netherlands and Sweden) [18]. For surgery‐related bleeds at index and post‐index, normal haemostasis was achieved after all bleeds where it was relevant.

The present findings are also in agreement with those of a retrospective analysis of real‐world experience of using rVWF in surgical procedures in French haemostasis centres [19]. For surgery‐related bleeds, patients received rVWF (with 11% also receiving recombinant FVIII) in that study. Physicians rated the haemostatic efficacy of rVWF as ‘good’ or ‘excellent’ in all patients, and the clinical efficacy as ‘good’ or ‘excellent’ in 97% of surgeries [19].

The outcomes of this real‐world chart review also are in line with those observed in a pivotal Phase 3 clinical trial with rVWF conducted in a surgical setting, in which bleed control was rated ‘good’ or ‘excellent’ for all bleeds that occurred within the trial setting [14]. However, in the present study, values for rVWF dose per infusion for bleeding episodes were lower on average than licensed recommendations [12], suggesting a difference in real‐world utilisation versus clinical trials. In this study, the average dose of rVWF was highest in prophylaxis in preparation for surgery and on the day of surgery, and then decreased post‐operatively, specifically when administered as prophylaxis following surgery, as would be expected.

Results from the stratified analyses demonstrated the variability of rVWF dosing across different patient characteristics, such as VWD type and surgery type. For example, higher doses were associated with major surgeries compared to minor or dental‐related surgeries. This highlights the importance of the assessment of the context and characteristics of each bleed event and the individual needs of the patient in providing the most appropriate regimen to prevent or resolve surgery‐related bleeding.

The limited available evidence indicates that HCRU is higher among people with VWD than in the general population. Lu et al. [11] determined that major bleeding events in patients with VWD were associated with increased HCRU and costs in the USA, primarily driven by inpatient costs as a result of more hospital admissions of longer duration, compared with patients without major bleeding. Furthermore, in a study of national registers in Sweden, Holm et al. [9] found that individuals with VWD consumed considerably more healthcare resources, with twice as many hospitalisations, compared with controls.

The results presented here are, to our knowledge, the first to report RWE of HCRU in UK patients with VWD who received rVWF to prevent or treat surgery‐related bleeds. The inpatient stay and outpatient visit rates at index rVWF administration to treat or prevent a surgery‐related bleed in this study (75.0% and 30.0%, respectively) were similar to those in Sun et al.'s [20] European study (75% and 26%, respectively). More research is required to assess HCRU in patients with VWD across treatment centres both within and outside the UK.

The study design enabled the inclusion of patients undergoing a range of surgery types across a diverse population. This provided evidence to support the versatility of use of rVWF in various surgical contexts in the real world whilst maximising the available sample size to facilitate confidence in the study results. However, the sample size was a limitation, given that the index rVWF treatments were separated across prophylaxis in preparation for surgery, treatment on the day of surgery (pre‐, intra‐ or post‐operatively), and/or prophylaxis following surgery, resulting in small numbers of patients at each stage. As a result, outliers may have considerably skewed the mean results presented, for example, in terms of dose or treatment duration. Furthermore, the limited sample size prohibited further stratification of the results by more specific types of surgery. Multiple treatments administered during the same surgery stage cannot be interpreted as simultaneous use of the products, as the exact timing of treatment administration was not recorded. Bleed severity, bleed control, bleed resolution and physician satisfaction were all subjective assessments and therefore subject to the inherent limitations of retrospective studies. Finally, almost all the adults with VWD treated with rVWF in a surgical setting had type 1 or 2 VWD, limiting the generalisability of these results to other VWD types. Further research is needed to address treatment patterns and outcomes for patients with type 3 VWD.

## 5 Conclusions

The results of this real‐world UK chart review add to the growing body of published evidence supporting the effectiveness and safety profile of rVWF for the prevention and management of surgery‐related bleeds in adults with type 1 and type 2 VWD. The real‐world nature of this study also provides valuable insights into how physicians prescribe and administer rVWF to their patients in clinical practice, and how this may differ from clinical trials and/or licensed recommendations.

## Author Contributions

Mike Laffan made substantial contributions to the conception and design of the work and acquisition of data for the work, and critically revised the work for important intellectual content. Heena Howitt made substantial contributions to the conception and design of the work and interpretation of data for the work, and critically revised the work for important intellectual content. Cheryl Jones made substantial contributions to the conception and design of the work and the analysis of data for the work, critically revised the work for important intellectual content. Sarah Brighton made substantial contributions to the analysis and interpretation of data for the work, and critically revised the work for important intellectual content. Rosa Willock made substantial contributions to the conception and design of the work and analysis of data for the work, and critically revised the work for important intellectual content. Anna Sanigorska made substantial contributions to the analysis of data for the work, and critically revised the work for important intellectual content. Oliver Heard made substantial contributions to the interpretation of data for the work and critically revised the work for important intellectual content. All authors approved the final version to be published and agreed to be accountable for all aspects of the work in ensuring that questions related to the accuracy or integrity of any part of the work are appropriately investigated and resolved.

## Conflicts of Interest

Mike Laffan has received grant/research support from LEO Pharma, Takeda, Pfizer, Roche‐Chugai, Sobi, AstraZeneca and BioMarin, has acted as a consultant for AstraZeneca on BMN331201 HAE gene therapy. Heena Howitt and Oliver Heard were employees of Takeda UK Ltd. at the time of this study; they are now ex‐employees. Sarah Brighton, Cheryl Jones, Rosa Willock and Anna Sanigorska were employees of HCD Economics, an organisation paid by Takeda UK Ltd. for contracted research, at the time of this study; they are now ex‐employees.

## Supporting information


**Data S1:** ejh70033‐sup‐0001‐Supinfo.docx.

## Data Availability

The data are not publicly available due to privacy or ethical restrictions. All data relevant to the study are included in the article or uploaded as Supporting Information.
