# Supplementary material for: Outcomes and Healthcare Resource Utilisation in Adults With von Willebrand Disease Treated With Recombinant von Willebrand Factor in Surgical Settings in the United Kingdom
Source: Eur J Haematol. 2025 Oct 11;116(1):75–84. doi: 10.1111/ejh.70033 (PMC12673352; doi:10.1111/ejh.70033)
Supplement: Supplementary file 1 — Data S1: ejh70033‐sup‐0001‐Supinfo.docx. [file EJH-116-75-s001.docx]

Outcomes and Healthcare Resource Utilisation in Adults with von Willebrand Disease Treated with Recombinant von Willebrand Factor in Surgical Settings in the United Kingdom

*Authors:* Mike Laffan^1^, Heena Howitt^2^, Cheryl Jones^3^, Sarah Brighton^3^, Rosa Willock^3^, Anna Sanigorska^3^, Oliver Heard^2^

*Author affiliations:*

^1^Centre for Haematology, Imperial College London, Hammersmith Hospital, London, UK

^2^Takeda UK Ltd., London, UK

^3^HCD Economics, Knutsford, UK

*Correspondence details:*

Name: Mike Laffan

Address: Centre for Haematology, Imperial College London

Hammersmith Hospital

London

W12 ONN

Telephone: +44 7788 106057

Email: [m.laffan@imperial.ac.uk](mailto:m.laffan@imperial.ac.uk)

**Supporting Information**

**Figure S1** A) Study design and B) timing of treatment administration

**A) Study design**

**B) Timing of treatment administration**

rVWF, recombinant von Willebrand factor.

**Table S1** Demographics and baseline characteristics of all adults who experienced a spontaneous or traumatic or surgery-related bleed that was treated with rVWF at index

| **Characteristic** | **N=32** |
| --- | --- |
| Age at index (years), mean (SD)  Age groups at index, n (%)  18–29 years  30–44 years  45–64 years  ≥65 years | 48.3 (19.5)  5 (15.6)  13 (40.6)  5 (15.6)  9 (28.1) |
| Female, n (%)  White/Caucasian, n (%) | 22 (68.8)  29 (90.6) |
| Weight (kg), mean (SD)  BMI (kg/m^2^), mean (SD)  VWD type, n (%)  Type 1  Type 2A  Type 2B  Type 2M  Type 2N  Unclassified  Age at diagnosis (years), mean (SD)  Time since diagnosis (years), mean (SD)  Familial history of VWD, n (%)  Unknown, n (%)  Family member with VWD, n (%)^†^  Parent  Sibling  Grandparent  Other  No history of GI bleeding, n (%)  Laboratory test values at diagnosis (IU/ml)^‡^, mean (SD)  VWF:RCo [n=18]  FVIII:C [n=18]  VWF:Ag [n=19] | 82.6 (18.6)  28.7 (5.5)  9 (28.1)  6 (18.8)  7 (21.9)  7 (21.9)  1 (3.1)  2 (6.3)  27.0 (20.3)  20.8 (15.8)  17 (53.1)  11 (34.4)  13 (76.5)  8 (47.1)  4 (23.5)  9 (52.9)  30 (93.8)  0.2 (0.2)  0.6 (0.3)  0.2 (0.1) |
| Comorbidities at index^§^, n (%)  0  1  2  >2  Comorbidities at index by type^¶^, n (%)  Disease of the musculoskeletal system or connective tissue  Circulatory  Disease of digestive system  Respiratory & ENT  Autoimmune  Mental or behavioural disorder  Oncology  Other  Cardiovascular  Endocrine, nutritional or metabolic disease  Neurological  Obstetrics/gynaecology  Disease of the blood/blood-forming organs  Disease of the genitourinary system  Liver disease  Ophthalmology | 13 (40.6)  6 (18.8)  4 (12.5)  9 (28.1)  8 (25.0)  6 (18.8)  6 (18.8)  4 (12.5)  3 (9.4)  3 (9.4)  3 (9.4)  3 (9.4)  2 (6.3)  2 (6.3)  2 (6.3)  2 (6.3)  1 (3.1)  1 (3.1)  1 (3.1)  1 (3.1) |

N=number of patients.

^†^Percentage calculated with number of patients with non-missing data in denominator.

^‡^Not all lab tests were performed at diagnosis on all patients. Mean (SD) are reported only for patients on whom each test was performed [n=x].

^§^Within two years of data abstraction.

^¶^At least one comorbidity in category.

Ag, antigen; BMI, body mass index; ENT, ear, nose and throat; FVIII, factor VIII; GI, gastrointestinal; RCo, ristocetin cofactor; rVWF, recombinant von Willebrand factor; SD, standard deviation; VWD, von Willebrand disease; VWF, von Willebrand factor.

**Table S2** Laboratory test values at presentation for adults who experienced a surgery-related bleed that was treated with rVWF at index by VWD type and by surgery type

|  | **VWF:RCo (IU/ml)** | **FVIII:C (IU/ml)** | **VWF:Ag (IU/ml)** |
| --- | --- | --- | --- |
| VWD type |  |  |  |
| Type 1 | 0.4 (0.3) [n=4] | 0.7 (0.5) [n=6] | 0.4 (0.3) [n=6] |
| Type 2 | 0.7 (0.8) [n=9] | 1.2 (0.8) [n=9] | 1.2 (1.0) [n=9] |
| Unclassified | 0.5 (0.5) [n=2] | 1.0 (0.4) [n=2] | 1,0 (02) [n=2] |
| Surgery type |  |  |  |
| Major | 0.7 (0.7) [n=10] | 1.2 (0.7) [n=10] | 1.2 (1.0) [n=10] |
| Minor | 0.3 (0.4) [n=5] | 0.6 (0.5) [n=7] | 0.6 (0.5) [n=7] |

All data are presented as mean (SD).

Not all lab tests were performed at presentation on all patients. Mean (SD) are reported only for patients on whom each test was performed [n=x].

Ag, antigen; FVIII, factor VIII; RCo, ristocetin cofactor; rVWF, recombinant von Willebrand factor; SD, standard deviation; VWD, von Willebrand disease; VWF, von Willebrand factor.

**Table S3** Treatment and prevention of surgery-related pre- and post-index bleeds in adults with VWD treated in a surgical setting

| **Variable** | **Pre-index (N=4 surgeries)** | | **Post-index (N=13 surgeries)** | | |
| --- | --- | --- | --- | --- | --- |
|  | **Prophylaxis in preparation for surgery (n=1)** | **As needed  on the day of surgery (n=3)** | **Prophylaxis in preparation for surgery (n=6)** | **As needed  on the day of surgery (n=7)** | **Prophylaxis to prevent further bleeds in the days following surgery (n=3)** |
| Treatment, n (%)  DDAVP only  Human VWF only  pdVWF/VIII complex only  TXA only  rVWF only  DDAPV + TXA  rVWF + TXA  pdVWF/FVIII complex + TXA | 1 (100.0)  –  –  –  –  –  –  – | –  1 (33.3)  1 (33.3)  1 (33.3)  –  –  –  – | –  –  –  –  2 (33.3)  1 (16.7)  1 (16.7)  2 (33.3) | –  –  –  –  7 (100.0)  –  –  – | –  –  –  –  1 (33.3)  –  1 (16.7)  1 (16.7) |
| DDAVP dosing (mcg), mean (SD)  Number of infusions  Dose per infusion (μg/kg)  Total consumption (μg)  Duration of treatment (days) | 20.0 (0.0)  1.0 (0.0)  0.3 (0.0)  20.0 (0.0)  1.0 (0.0) | –  –  –  –  – | 15.0 (0.0)  1.0 (0.0)  0.3 (0.0)  15.0 (0.0)  1.0 (0.0) | –  –  –  –  – | –  –  –  –  – |
| Human VWF dosing (IU), mean (SD)  Number of infusions  Dose per infusion (IU/kg)  Total consumption (IU)  Duration of treatment (days) | –  –  –  –  – | 3000.0 (0.0)  1.0 (0.0)  46.9 (0.0)  3000.0 (0.0)  1.0 (0.0) | –  –  –  –  – | –  –  –  –  – | –  –  –  –  – |
| pdVWF/FVIII complex dosing (IU), mean (SD)  Number of infusions  Dose per infusion (IU/kg)  Total consumption (IU)  Duration of treatment (days) | –  –  –  –  – | 3000.0 (0.0)  1.0 (0.0)  33.7 (0.0)  3000.0 (0.0)  1.0 (0.0) | 2500.0 (707.1)  1.0 (0.0)  37.0 (13.0)  2500.0 (707.1)  1.0 (0.0) | –  –  –  –  – | 2000.0 (0.0)  1.0 (0.0)  27.8 (0.0)  2000.0 (0.0)  1.0 (0.0) |
| TXA dosing (mg), mean (SD)  Number of infusions  Dose per infusion (mg/kg)  Total consumption (mg)  Duration of treatment (days) | –  –  –  –  – | 1000.0 (0.0)  1.0 (0.0)  12.3 (0.0)  1000.0 (0.0)  1.0 (0.0) | 1000.0 (0.0)  6.3 (6.7)  14.5 (3.5)  6250.0 (6702.0)  2.3 (1.9) | –  –  –  –  – | 1000.0 (0.0)  35.0 (9.9)  12.0 (2.7)  35,000.0 (9899.5)  24.5 (24.7) |
| rVWF dosing (IU), mean (SD)  Number of infusions  Dose per infusion (IU/kg)  Total consumption (IU)  Duration of treatment (days) | –  –  –  –  – | –  –  –  –  – | 3250.0 (1125.8)  1.0 (0.0)  35.1 (9.4)  3250.0 (1125.8)  1.0 (0.0) | 2878.6 (1629.6)  1.1 (0.4)  29.3 (12.1)  3157.1 (1611.0)  1.1 (0.4) | 2080.0 (1654.6)  8.0 (2.8)  21.7 (15.7)  14,300.0 (7353.9)  4.5 (0.7) |

DDAVP, desmopressin; FVIII, factor VIII; pd, plasma-derived; rVWF, recombinant von Willebrand factor; SD, standard deviation; TXA, tranexamic acid; VWF, von Willebrand factor; VWD, von Willebrand disease.
